# Supplementary figures and images for: Natural Selection Reduced Diversity on Human Y Chromosomes
Source: PLoS Genet. 2014 Jan 9;10(1):e1004064. doi: 10.1371/journal.pgen.1004064 (PMC3886894; doi:10.1371/journal.pgen.1004064)

# Y diversity corrected for different divergence

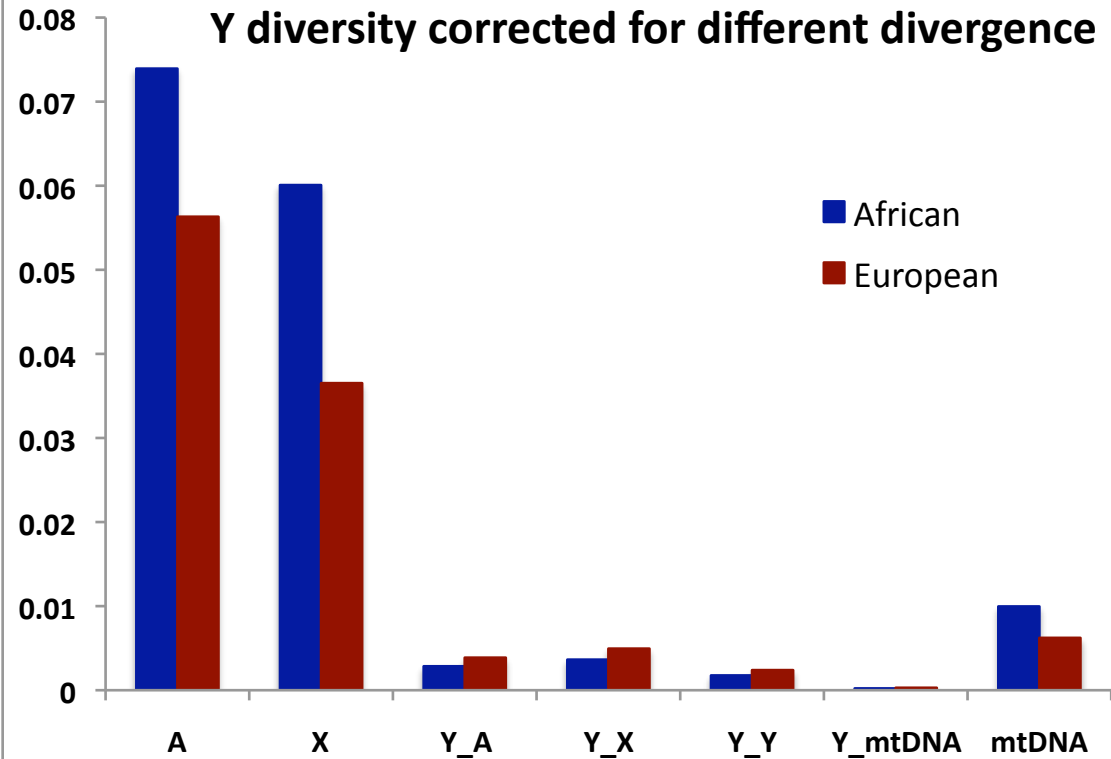

Supplement: Figure S1 — Divergence on the Y chromosome. If we are overcorrecting for human-chimpanzee divergence on the Y chromosome, then we may under-estimate normalized Y diversity. To assess how different divergence corrections affect the normalized diversity estimate on the Y, we corrected raw Y diversity by divergence computed for the autosomes (Y_A), the X chromosome (Y_X), the Y chromosome (Y_Y) and the mtDNA (Y_mtDNA). In all cases, normalized diversity on the Y chromosome is still extremely low. (PDF) [file pgen.1004064.s001.pdf]

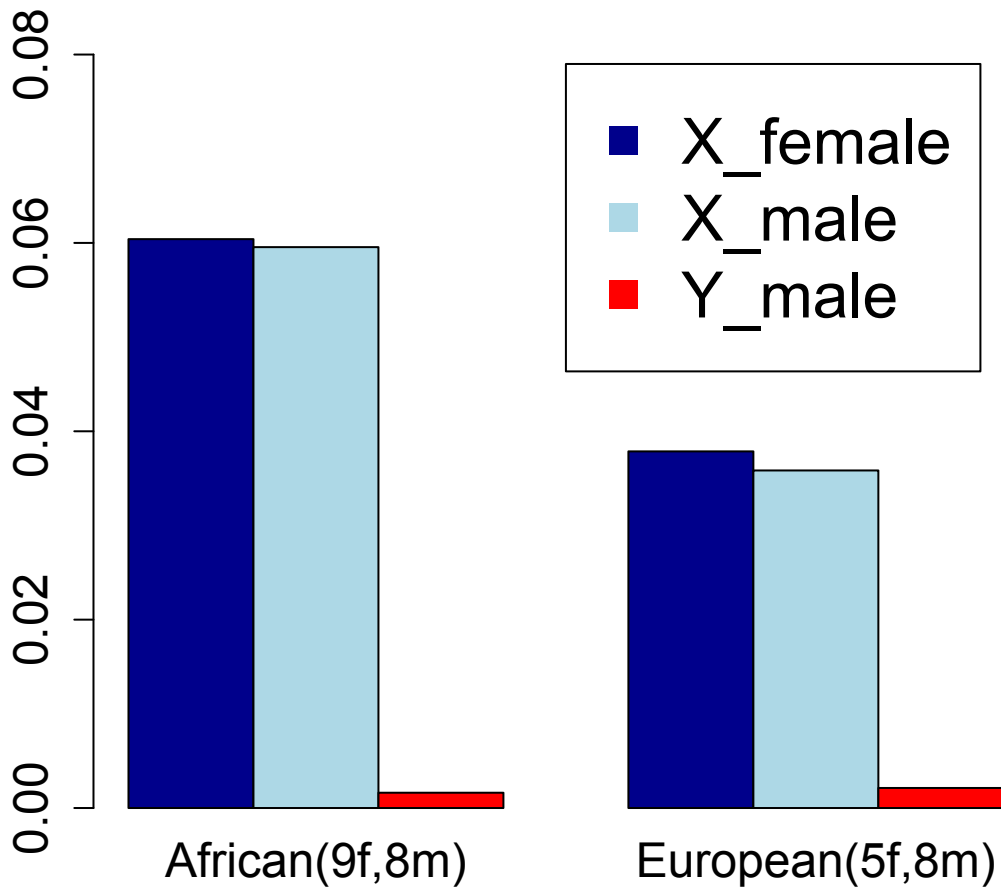

Supplement: Figure S2 — Diploid versus haploid calling. If Complete Genomics is under-calling variation on the Y chromosome due to its haploid nature, then we would expect it to also under-call variation on the X chromosome in males. So, we compared diversity on the X chromosome, when computed across the unrelated African and European males (as presented in the main text) with diversity on the X as computed in the unrelated African and European females, and find that there are no large reductions in diversity on the X, when using males, m, (where the X is haploid) versus females, f, (where the X is diploid). (PDF) [file pgen.1004064.s002.pdf]

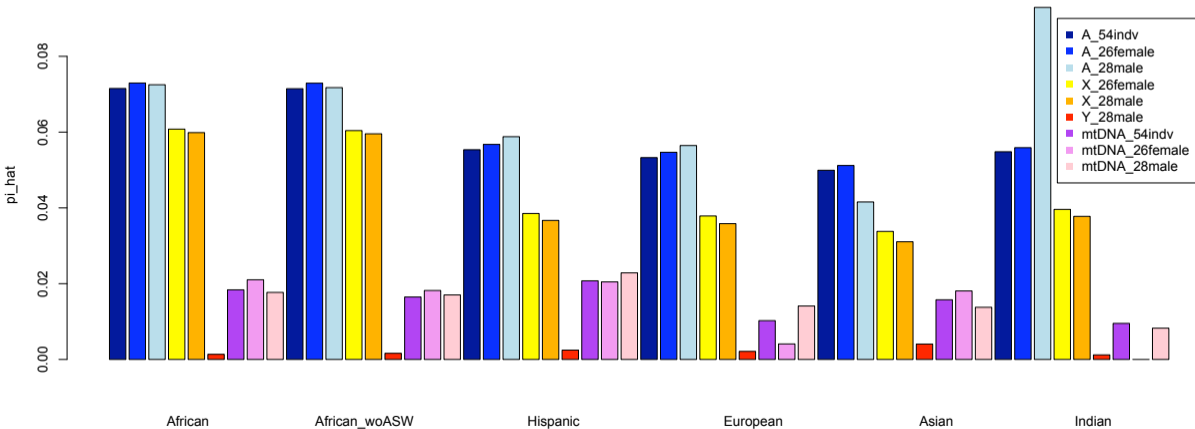

Supplement: Figure S3 — Diversity across populations. All major populations from the Complete Genomics data were initially analyzed (African, African without African Americans, Hispanic, European, East Asian and Indian), using both the male and the female samples for all chromosome regions (Autosomes, Chromosome X, Chromosome Y and mtDNA. Where possible we analyzed all individuals from the subpopulation regardless of gender (analyzing all autosomes from the populations available from the complete set of 54 individuals), or analyzed diversity on the X and mtDNA across all females from the subpopulation from the 26 unrelated females, or analyzed diversity on the X, mtDNA and Y across all males from the subpopulation from the 28 unrelated males. In every population, diversity on the Y was noticeably reduced. (PDF) [file pgen.1004064.s003.pdf]

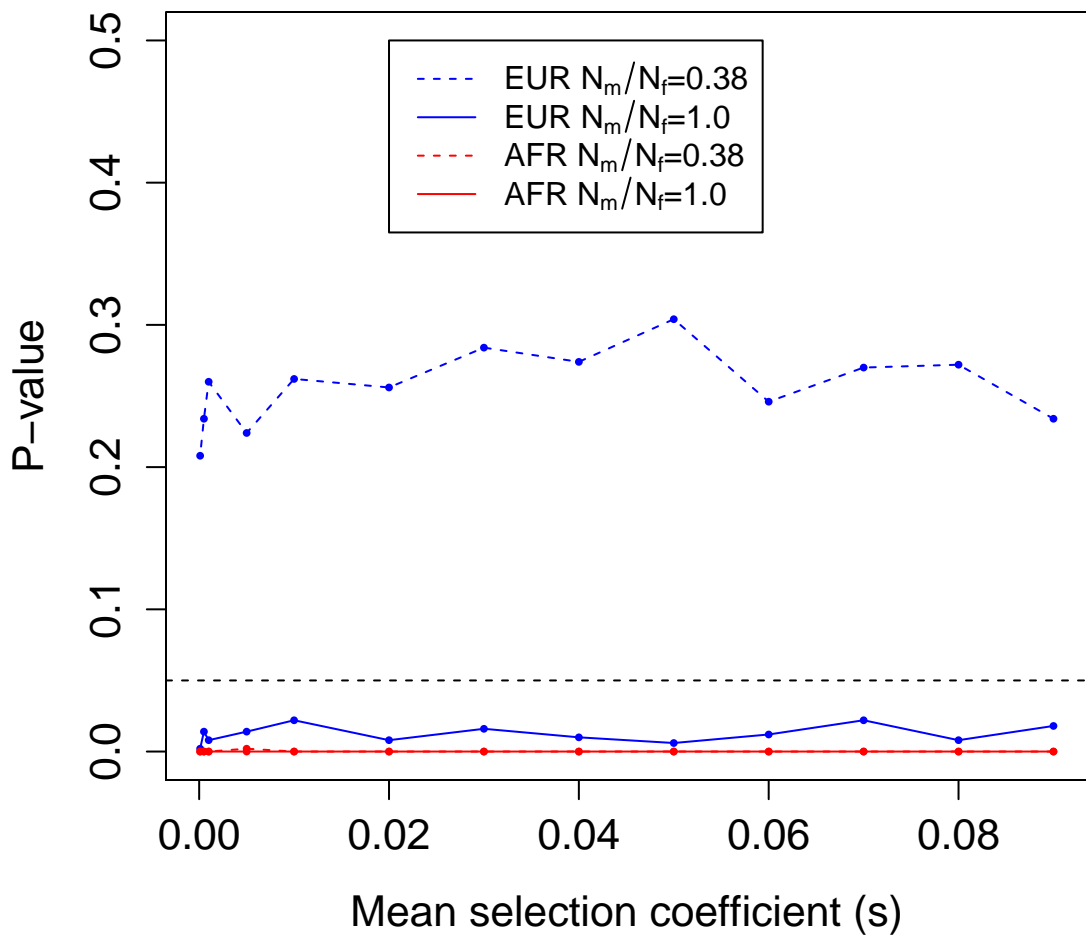

Supplement: Figure S4 — Purifying selection on coding sites. Models of selection acting only on coding sites cannot sufficiently reduce expected diversity on the Y chromosome. Under assumptions of equal sex ratios (N m/N f = 1), simulations using different mean selection coefficients (s), result in expectations of diversity that are significantly different from the observed values for both Africans and Europeans. Alternatively, under an assumption of an extremely reduced male effective population size, relative to females (N m/N f = 0.38), diversity is still significantly different from observations in Africans, but passes into a range of being consistent with observed diversity in Europeans. We fixed the shape parameter to 0.184 and performed simulations using mean selection coefficients ranging from 0.0001 to 0.09. (PDF) [file pgen.1004064.s004.pdf]

Comparison of branch lengths

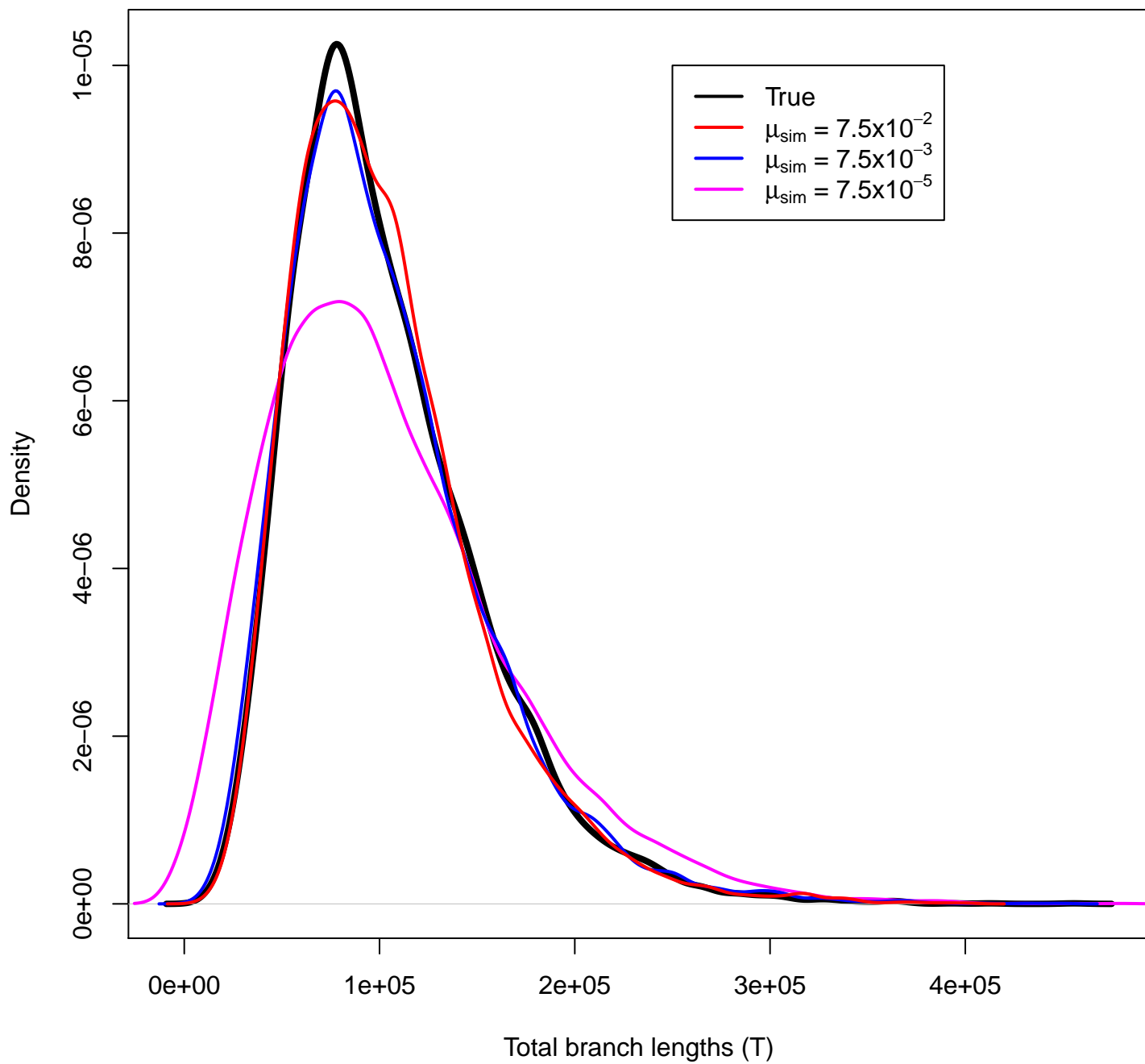

Supplement: Figure S5 — Branch length comparisons. Total tree length cannot easily be estimated from forward simulations with multiple sites under selection but can be approximated by the number of segregating sites. Comparisons are shown of the distribution of total branch lengths (in units of generations) for genealogies of 8 chromosomes from a population of size 10,000 diploids simulated under the standard neutral model without recombination using ms. The black curve denotes the actual distribution of total branch length for all of the 10,000 simulated genealogies. The remaining curves show the distribution of branch lengths estimated by dividing the number of segregating sites by μsim. As μsim becomes large (>0.0075), the distribution of branch lengths inferred from the number of segregating sites approaches the true distribution. In other words, the majority of variance in the number of segregating sites is due to the variance of the total branch lengths, rather than the Poisson variance in the number of mutations conditional on the given genealogy. As such, the number of segregating sites in simulations can be taken as a reasonable proxy for the total branch lengths of the genealogy. (PDF) [file pgen.1004064.s005.pdf]

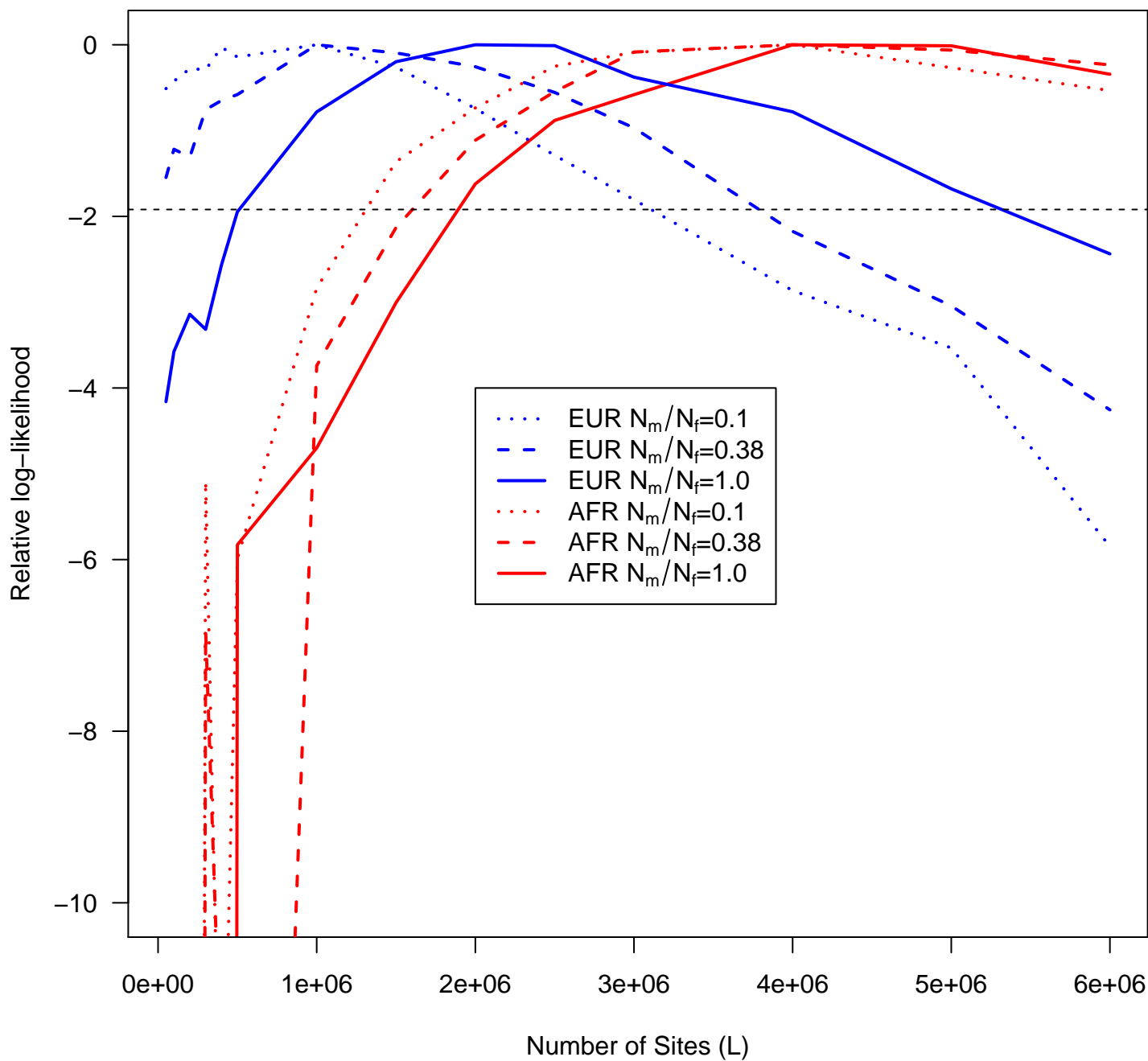

Supplement: Figure S6 — Log-likelihood curves for the number of sites affected by purifying selection (L). Blue curves are from the European population and red curves are from the Africans. Dotted lines denote sex-biased demography where N m/N f = 0.1, the dashed lines denote sex-biased demography where N m/N f = 0.38 while the solid lines represent equal sex ratios (N m/N f = 1). The horizontal dashed line denotes the asymptotic 95% confidence interval cutoff (1.92 log-likelihood units), such that values above this line result in estimates of diversity that are consistent with observations. (PDF) [file pgen.1004064.s006.pdf]

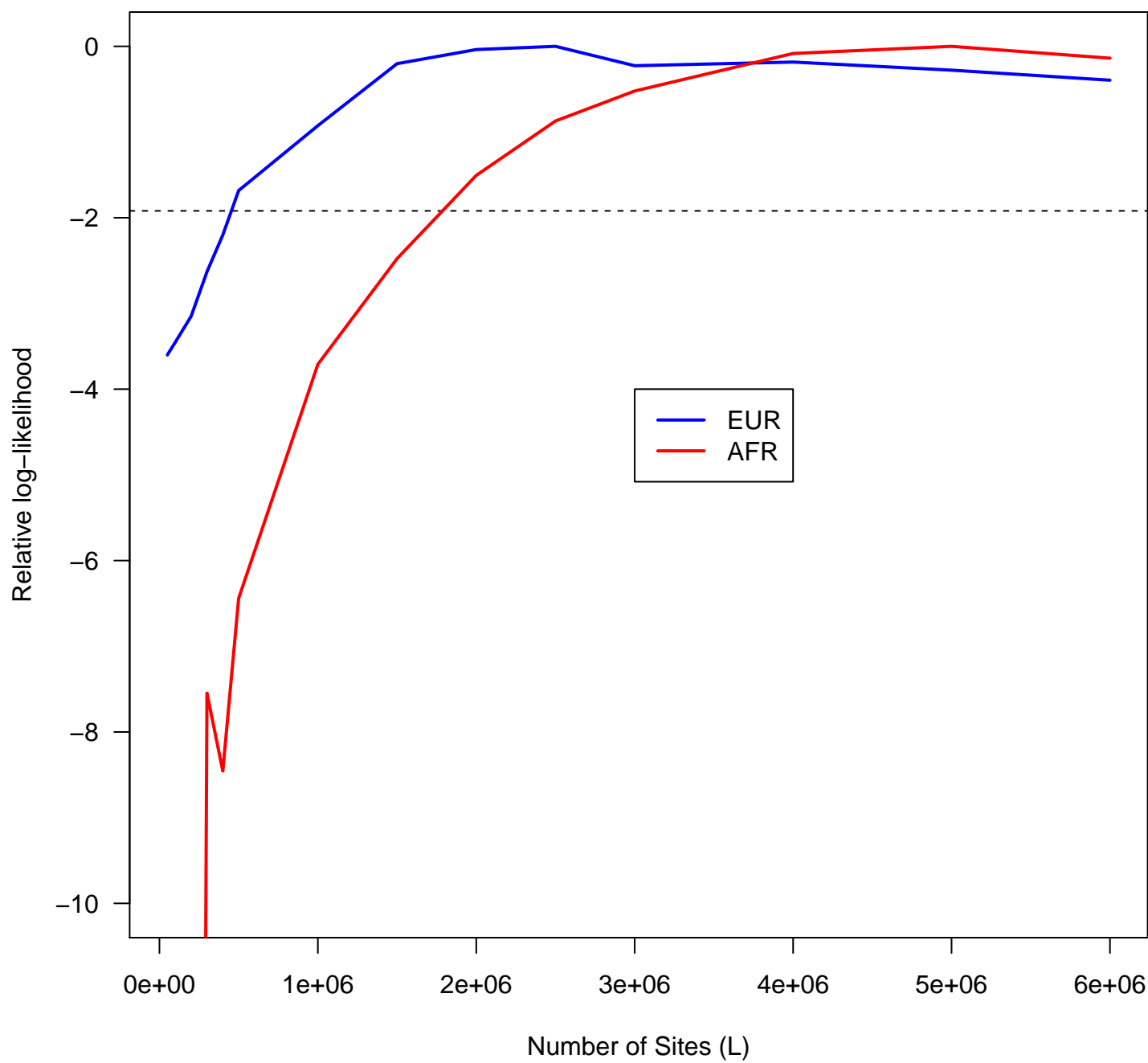

Supplement: Figure S7 — Profile log-likelihood curves for the number of sites affected by purifying selection (L) when jointly estimating L and the mean strength of selection. Blue curves are from the European population and red curves are from the Africans. The horizontal dashed line denotes the asymptotic 95% confidence interval cutoff (1.92 log-likelihood units), such that values above this line result in estimates of diversity that are consistent with observations. (PDF) [file pgen.1004064.s007.pdf]

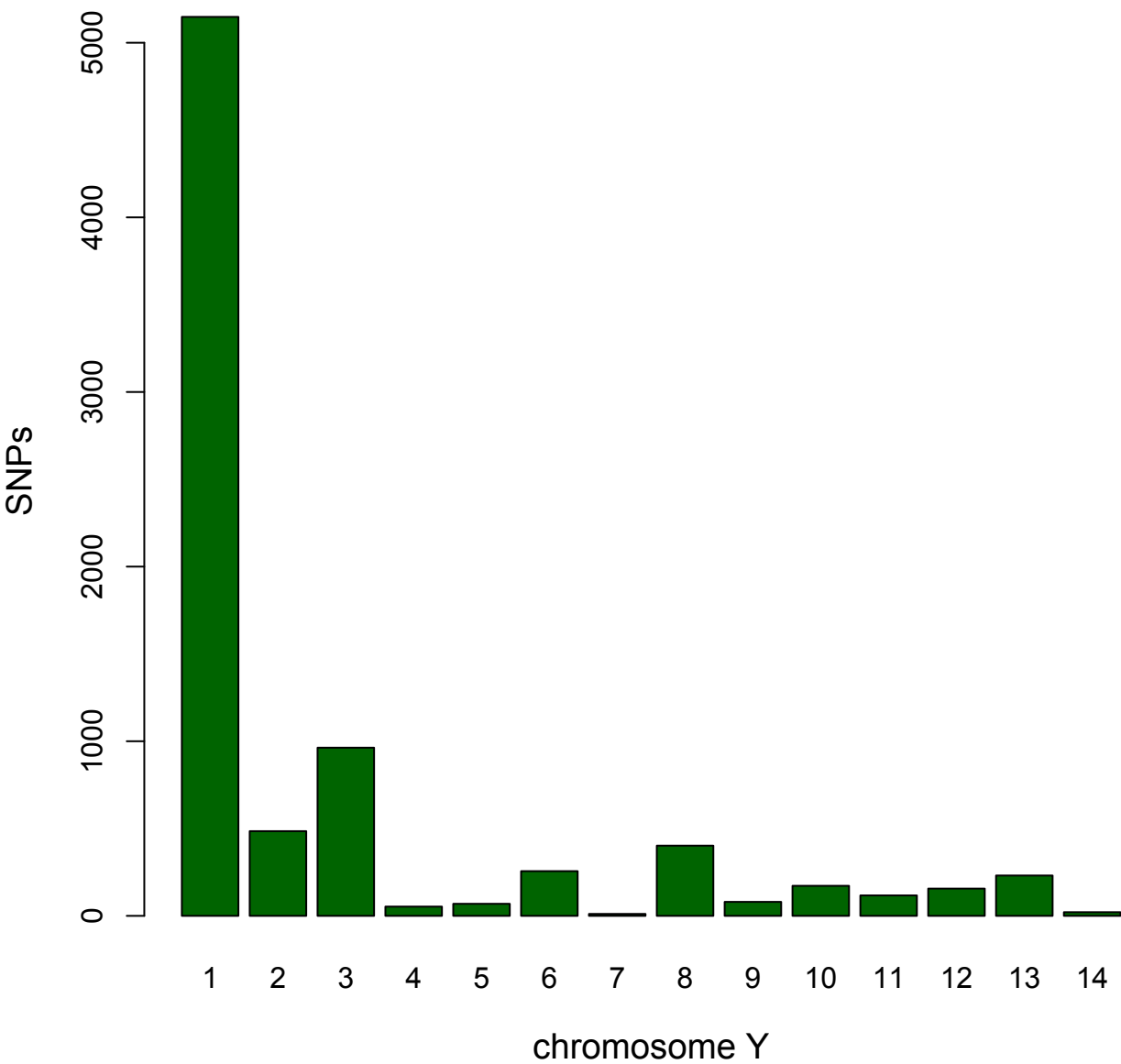

Supplement: Figure S8 — Y chromosome site frequency spectrum. The folded site frequency spectrum for Y chromosome SNPs across all 28 unrelated Y chromosomes in the Complete Genomics dataset is shown below. We include all unrelated males to limit reduce stochastic variation from analyzing only the 8 African and 8 Europeans of the main text. The abundance of low frequency SNPs is consistent with both positive selection and purifying selection. Given the very low total number of SNPs on the human Y chromosome, and the high divergence between human and chimpanzee, we only analyzed the folded site frequency spectrum. (PDF) [file pgen.1004064.s008.pdf]

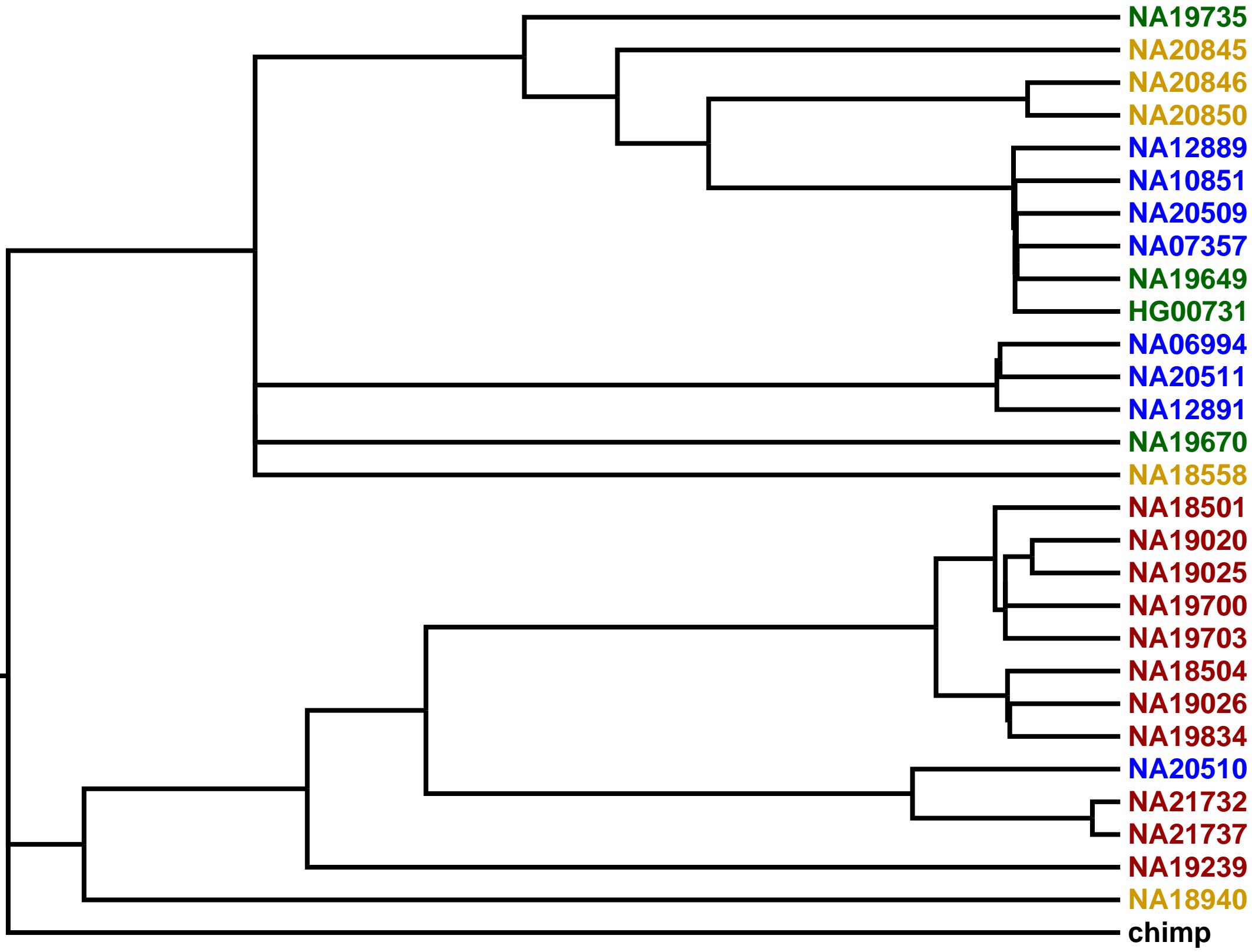

0.02

Supplement: Figure S9 — Y chromosome haplotype tree. A neighbor-joining tree was built for all unrelated Y haplotypes in the Complete Genomics dataset in phylip [50], then branch lengths were computed using a molecular clock in paml [51]. We include all 28 males to gain a higher resolution into the topology of the phylogenetic tree. There is not an overarching star phylogeny, which would be indicative of positive selection. However, one might conceive of a complex evolutionary history involving several instances of positive selection along different Y lineages that could result in the observed haplotype topology. The colors correspond to reported population ancestry: red names correspond to individuals reported to be of African or African American descent, yellow corresponds to individuals reported to be of Asian descent, blue corresponds to people reported to be of European descent, and green corresponds to people reported to be of Latino descent. (PDF) [file pgen.1004064.s009.pdf]
