# Supplementary material for: Natural Selection Reduced Diversity on Human Y Chromosomes
Source: PLoS Genet. 2014 Jan 9;10(1):e1004064. doi: 10.1371/journal.pgen.1004064 (PMC3886894; doi:10.1371/journal.pgen.1004064)
Supplement: Note S1 — Simulation command line codes. Command lines for coalescent and forward simulations are described in detail. (DOCX) [file pgen.1004064.s010.docx]

**Natural selection reduced diversity on human Y chromosomes**

Melissa A. Wilson Sayres, Kirk E. Lohmueller, and Rasmus Nielsen

**Note S1. Simulation command line codes**

*Simulation command line codes*

Neutral expectations under equal and skewed sex ratios were modeled using coalescent simulations implemented in ms [^1^](#_ENREF_1), assuming the population-specific demographic models described above, and allowing for recombination on the autosomes and X chromosome, but not the Y or mtDNA (Methods; Table S7). The ms commands used take the forms below:

For Africans:

A & X ./ms *$indv* *$repl* -t *$theta* -r *$rho $sites* -eN *$time1 $size1*

Y & M ./ms *$indv* *$repl* -t *$theta* -r *$sites* -eN *$time1 $size1*

For Europeans:

A & X ./ms *$indv $repl* -t *$theta* -r *$rho $sites* -eN *$time1 $size1* -eN *$time2 $size2*

Y & M ./ms *$indv $repl* -t *$theta* -eN *$time1 $size1* -eN *$time2 $size2*

Where:

$indv = 16 for autosomes; 8 for X, Y and mtDNA;

$repl = 10000;

$theta = 4×$N×$u×$sites;

$rho = 4×$N×$r×$sites;

$time = African: $time1 = 4000/(4*$N);

European: $time1 = 1100/(4*$N);

$time2 = 1500/(4*$N);

$size = African: $size1 = 0.5;

European: $size1 = 0.01;

$size2 = 1;

We modeled purifying selection using forward simulations implemented in SFS_CODE [^2^](#_ENREF_2). To investigate purifying selection acting only on new nonsynonymous mutations, we simulated 60,041 nonsynonymous sites (2/3 the 90,062 coding sites estimated on the Y chromosome [^3^](#_ENREF_3)) at which new mutations are expected to be subject to purifying selection. To assess the effect of background selection, each simulation also contained 500kb of linked neutral sequence from which we calculated diversity. The SFS_CODE commands for modeling purifying selection on the coding sites are:

For Africans:

./sfs_code 1 50 -r 0 -t 0.000170954 -P 1 -TE 0.8 -Td 0 2.0 -L 2 500000 60041 -a N -W L 1 2 0 1 1 0.184 $ln -n 8 -N 500 –A

For Europeans:

./sfs_code 1 125 -r 0 -t 0.000170954 -P 1 -TE 0.3 -Td 0 0.1 -Td 0.08 10 -L 2 500000 60041 -a N -W L 1 2 0 1 1 0.184 $ln -n 8 -N 500 -A

Where, in each simulation, $ln=0.184/($mean_s*5000).

To estimate the number of sites affected by purifying selection on the Y chromosome (defined as *L*), we employ an approximate likelihood approach [^4-6^](#_ENREF_4) using the observed number of segregating sites as a summary statistic. The SFS_CODE commands used to estimate the number of sites under selection are:

For Africans:

./sfs_code 1 50 -r 0 -t 0.000170954 -P 1 -TE 0.8 -Td 0 2.0 -L 2 500000 $L -a N -W L 1 2 0 1 1 0.184 0.000625272494772688 -n 8 -N 500 -A

For Europeans:

./sfs_code 1 125 -r 0 -t 0.000170954 -P 1 -TE 0.3 -Td 0 0.1 -Td 0.08 10 -L 2 500000 $L -a N -W L 1 2 0 1 1 0.184 0.000625272494772688 -n 8 -N 500 -A

$L is the number of sites subjected to purifying selection.
