# Supplementary material for: Natural Selection Reduced Diversity on Human Y Chromosomes
Source: PLoS Genet. 2014 Jan 9;10(1):e1004064. doi: 10.1371/journal.pgen.1004064 (PMC3886894; doi:10.1371/journal.pgen.1004064)
Supplement: Table S1 — Complete Genomics unrelated male samples. IDs, sex, population, ethnicity, and abbreviations are provided for each of the Complete Genomics samples used. We cross-checked each individual to make sure there were no previously unreported relationships between them that might confound analyses [75]. (DOCX) [file pgen.1004064.s011.docx]

| ID | Sex | Population | Ethnicity | Abbreviation |
| --- | --- | --- | --- | --- |
| NA18501 | Male | African | YORUBA | YRI |
| NA18504 | Male | African | YORUBA | YRI |
| NA19020 | Male | African | LUHYA | LWK |
| NA19025 | Male | African | LUHYA | LWK |
| NA19026 | Male | African | LUHYA | LWK |
| NA19239 | Male | African | YORUBA | YRI |
| NA21732 | Male | African | MAASAI | MKK |
| NA21737 | Male | African | MAASAI | MKK |
| NA06994 | Male | European | UTAH/MORMON | CEU |
| NA07357 | Male | European | UTAH/MORMON | CEU |
| NA10851 | Male | European | UTAH/MORMON | CEU |
| NA12889 | Male | European | UTAH/MORMON | CEU |
| NA12891 | Male | European | UTAH/MORMON | CEU |
| NA20509 | Male | European | TOSCANI (TUSCAN) | TSI |
| NA20510 | Male | European | TOSCANI (TUSCAN) | TSI |
| NA20511 | Male | European | TOSCANI (TUSCAN) | TSI |
