# Supplementary material for: Natural Selection Reduced Diversity on Human Y Chromosomes
Source: PLoS Genet. 2014 Jan 9;10(1):e1004064. doi: 10.1371/journal.pgen.1004064 (PMC3886894; doi:10.1371/journal.pgen.1004064)
Supplement: Table S2 — Uncorrected diversity (π) within Africans and Europeans, and human divergence from chimpanzee. All values are per site. Estimates of the mutation rate for the mtDNA are corrected for multiple substitutions using the Tamura-Nei model. (DOCX) [file pgen.1004064.s012.docx]

| Class | A | X | Y | mtDNA |
| --- | --- | --- | --- | --- |
| Africa diversity | 0.00093621 | 0.00059546 | 0.00003603 | 0.00169340 |
| European diversity | 0.00071314 | 0.00036196 | 0.00004901 | 0.00105811 |
| Sites in all 8 Africans | 1238501316 | 56363307 | 7758906 | 3004 |
| Sites in all 8 Europeans | 1246322049 | 56573306 | 7974045 | 3037 |
|  |  |  |  |  |
| Divergence from chimpanzee | 0.012664107 | 0.009911943 | 0.020514487 | 0.0720 |
