# Supplementary material for: Natural Selection Reduced Diversity on Human Y Chromosomes
Source: PLoS Genet. 2014 Jan 9;10(1):e1004064. doi: 10.1371/journal.pgen.1004064 (PMC3886894; doi:10.1371/journal.pgen.1004064)
Supplement: Table S4 — Observed and mean modeled ratios of neutral diversity under various estimates of the N m/N f ratio. For Africans, the neutral model is of an expansion from 10,000 individuals to 20,000 individuals 4,000 generations ago. For Europeans the neutral model includes a bottleneck from 10,000 individuals to 1,000 individuals 1,500 generations ago, followed by an expansion to 10,000 individuals 1,100 generations ago. Mean estimates from 10,000 simulations under various assumptions of the ratio of the effective number of males to the effective number of females (N m/N f) are shown for Autosomes (A), chromosome X, chromosome Y and mtDNA. (DOCX) [file pgen.1004064.s014.docx]

|  |  |  |  | *N*_m_/*N*_f_ | | | | | |
| --- | --- | --- | --- | --- | --- | --- | --- | --- | --- |
| Pop | Ratio | *Observed* |  | 1 | 0.9 | 0.75 | 0.5 | 0.38 | 0.25 |
| African | X/A | *0.8133* |  | 0.7732 | 0.7854 | 0.8091 | 0.8595 | 0.8962 | 0.9475 |
|  | Y/A | *0.0244* |  | 0.3038 | 0.2933 | 0.2725 | 0.2429 | 0.2291 | 0.2110 |
|  | mt/A | 0.3181 |  | 0.3053 | 0.3209 | 0.3439 | 0.4276 | 0.5006 | 0.6673 |
| European | X/A | *0.6483* |  | 0.7210 | 0.7383 | 0.7644 | 0.8287 | 0.8720 | 0.9355 |
|  | Y/A | *0.0426* |  | 0.1813 | 0.1681 | 0.1510 | 0.1265 | 0.1142 | 0.1025 |
|  | mt/A | *0.2610* |  | 0.1813 | 0.1965 | 0.2194 | 0.3027 | 0.3922 | 0.5802 |
