# Supplementary material for: Natural Selection Reduced Diversity on Human Y Chromosomes
Source: PLoS Genet. 2014 Jan 9;10(1):e1004064. doi: 10.1371/journal.pgen.1004064 (PMC3886894; doi:10.1371/journal.pgen.1004064)
Supplement: Table S5 — Performance of our approximate likelihood approach to estimate L on simulated data. L represents the number of sites affected by purifying selection. We assessed the accuracy of estimates by first making 1000 test datasets with a known value of L (L = 2 Mb). We then computed the maximum likelihood estimate (MLE) of the number of sites affected by selection using our approximate likelihood approach. The table shows the mean and medians of the MLEs over the 1000 test datasets for each model. We also recorded the percentage of asymptotic 95% confidence intervals that contained the true value of L. The results, summarized in the table below, show that our method can accurately estimate the number of sites affected by purifying selection. (DOCX) [file pgen.1004064.s015.docx]

|  | Mean MLE | Median MLE | % of 95% CIs that contain the true *L* |
| --- | --- | --- | --- |
| African | 2.23 Mb | 2.00 Mb | 96.6% |
| European | 2.24 Mb | 2.00 Mb | 98.3% |
