# Supplementary material for: Natural Selection Reduced Diversity on Human Y Chromosomes
Source: PLoS Genet. 2014 Jan 9;10(1):e1004064. doi: 10.1371/journal.pgen.1004064 (PMC3886894; doi:10.1371/journal.pgen.1004064)
Supplement: Table S6 — European observed and mean modeled estimates of diversity for various intensities of the population bottleneck. The model is of neutral evolution with a bottleneck from 1500 generations ago to 1100 generations ago, from an ancestral size of 10,000 individuals, and a contemporary size of 10,000 individuals. The size of the bottleneck is varied in the table below. A slightly less severe bottleneck (1000 versus 550) was chosen for our analyses because it was more consistent with the observed genome-wide autosomal data. (DOCX) [file pgen.1004064.s016.docx]

|  |  | Modeled diversity with varying bottlenecks | | |
| --- | --- | --- | --- | --- |
| Diversity | Observed | 550 individuals | 1000 individuals | 2000 individuals |
| Autosome | 0.0563 | 0.04832 | 0.05633 | 0.06196 |
| chrX | 0.0365 | 0.03330 | 0.03992 | 0.04541 |
| chrY | 0.0024 | 0.00695 | 0.01008 | 0.01299 |
| mtDNA | 0.0147 | 0.00682 | 0.01005 | 0.01302 |
