# Supplementary material for: Natural Selection Reduced Diversity on Human Y Chromosomes
Source: PLoS Genet. 2014 Jan 9;10(1):e1004064. doi: 10.1371/journal.pgen.1004064 (PMC3886894; doi:10.1371/journal.pgen.1004064)
Supplement: Table S7 — Comparing chromosome-wide SNPs. In an effort to determine whether the Complete Genomics dataset is dramatically under-calling SNPs on the Y chromosome, we compared the total number of sites and SNPs in the set of sixteen male samples that overlap between the unrelated males in the Complete Genomics public dataset, and the 1000 genomes dataset: NA19700 (ASW), NA19703 (ASW), NA19834 (ASW), NA18501 (YRI), NA18504 (YRI), NA19020 (LWK), HG00731 (PUR), NA19735 (MXL), NA20509 (TSI), NA20510 (TSI), NA06994 (CEU), NA07357 (CEU), NA10851 (CEU), NA12889 (CEU), NA18558 (CHB), and NA18940 (JPT). We find that, whether we use no filtering (SNPs called on the Y in any 16 males, regardless of whether the sites were called in any other individual), or the same conservative filtering applied in the main manuscript (requiring that sites be called in all individuals analyzed), there are many more SNPs called in the Complete Genomics dataset, than in the 1000 genomes. This cannot be attributed to different amounts of sequences assayed, as both assay roughly 22 Mb of sequence on the Y chromosome. Because the 1000 genomes does not report sites called for each individual, we report data from the one mask file they share, which is sites called across all individuals. For the Complete Genomics data we report both filters of the total number of sites assayed (either called in any individual, or requiring the site be called in all). (DOCX) [file pgen.1004064.s017.docx]

| Filtering | Complete Genomics | 1000 Genomes | Sites called in 1000 genomes, and not CG |
| --- | --- | --- | --- |
| SNPs in any 16 males | 13197 | 2136 | 213 (all singletons) |
| SNPs in all 16 males | 6236 | 252 | 12 (all singletons) |
| Total sites | 22848852*/20480009** | 22984529 | - |
| *Sites in any individual | | | |
| **Sites in all individuals | | | |
