# Supplementary material for: Natural Selection Reduced Diversity on Human Y Chromosomes
Source: PLoS Genet. 2014 Jan 9;10(1):e1004064. doi: 10.1371/journal.pgen.1004064 (PMC3886894; doi:10.1371/journal.pgen.1004064)
Supplement: Table S8 — Parameters used in ms simulations. For all simulations, African: size1 = 0.5, and European: size1 = 0.01, and size2 = 1. The models for Africans assume an expansion from 10,000 to 20,000 individuals, 4,000 generations ago, and for Europeans a bottleneck from 1,500 to 1,100 generations ago, from an ancestral size of 10,000 individuals, reduced to 1,000, then expanded back to 10,000 individuals. As the ratio of the effective number of males and females (N m/N f) varies, the effective population size of the autosomes (A) is held constant. (DOCX) [file pgen.1004064.s018.docx]

|  |  | Africans | | | |  | Europeans | | | | |
| --- | --- | --- | --- | --- | --- | --- | --- | --- | --- | --- | --- |
| Chr | *N*_m_/*N*_f_ | *N*_o_ | theta | rho | time1 |  | *N*_o_ | theta | rho | time1 | time2 |
| A | 1 | 20000.00 | 1688.54 | 800.00 | 0.0500 |  | 10000.00 | 844.27 | 400.00 | 0.0275 | 0.0375 |
| X | 1 | 15000.00 | 991.19 | 400.00 | 0.0667 |  | 7500.00 | 495.60 | 200.00 | 0.0367 | 0.0500 |
| X | 0.9 | 15267.86 | 1008.89 | 407.14 | 0.0655 |  | 7633.93 | 504.45 | 203.57 | 0.0360 | 0.0491 |
| X | 0.75 | 15750.00 | 1040.75 | 420.00 | 0.0635 |  | 7875.00 | 520.38 | 210.00 | 0.0349 | 0.0476 |
| X | 0.5 | 16875.00 | 1115.09 | 450.00 | 0.0593 |  | 8437.50 | 557.55 | 225.00 | 0.0326 | 0.0444 |
| X | 0.38 | 17642.05 | 1165.78 | 470.45 | 0.0567 |  | 8821.02 | 582.89 | 235.23 | 0.0312 | 0.0425 |
| X | 0.25 | 18750.00 | 1238.99 | 500.00 | 0.0533 |  | 9375.00 | 619.50 | 250.00 | 0.0293 | 0.0400 |
| X | 0.1 | 20625.00 | 1362.89 | 550.00 | 0.0485 |  | 10312.50 | 681.45 | 275.00 | 0.0267 | 0.0364 |
| Y | 1 | 5000.00 | 5305.66 | - | 0.2000 |  | 2500.00 | 2652.83 | - | 0.1100 | 0.1500 |
| Y | 0.9 | 4750.00 | 5040.38 | - | 0.2105 |  | 2375.00 | 2520.19 | - | 0.1158 | 0.1579 |
| Y | 0.75 | 4375.00 | 4642.46 | - | 0.2286 |  | 2187.50 | 2321.23 | - | 0.1257 | 0.1714 |
| Y | 0.5 | 3750.00 | 3979.25 | - | 0.2667 |  | 1875.00 | 1989.62 | - | 0.1467 | 0.2000 |
| Y | 0.38 | 3450.00 | 3660.91 | - | 0.2899 |  | 1725.00 | 1830.45 | - | 0.1594 | 0.2174 |
| Y | 0.25 | 3125.00 | 3316.04 | - | 0.3200 |  | 1562.50 | 1658.02 | - | 0.1760 | 0.2400 |
| Y | 0.1 | 2750.00 | 2918.12 | - | 0.3636 |  | 1375.00 | 1459.06 | - | 0.2000 | 0.2727 |
| M | 1 | 5000.00 | 6.17 | - | 0.2000 |  | 2500.00 | 3.09 | - | 0.1100 | 0.1500 |
| M | 0.9 | 5277.78 | 6.51 | - | 0.1895 |  | 2638.89 | 3.26 | - | 0.1042 | 0.1421 |
| M | 0.75 | 5833.33 | 7.20 | - | 0.1714 |  | 2916.67 | 3.60 | - | 0.0943 | 0.1286 |
| M | 0.5 | 7500.00 | 9.26 | - | 0.1333 |  | 3750.00 | 4.63 | - | 0.0733 | 0.1000 |
| M | 0.38 | 9078.95 | 11.21 | - | 0.1101 |  | 4539.47 | 5.60 | - | 0.0606 | 0.0826 |
| M | 0.25 | 12500.00 | 15.43 | - | 0.0800 |  | 6250.00 | 7.71 | - | 0.0440 | 0.0600 |
| M | 0.1 | 27500.00 | 33.94 | - | 0.0364 |  | 13750.00 | 16.97 | - | 0.0200 | 0.0273 |
